# Research and Implementation of Localization of Multiple Local Discharge Sources in Switchgear Based on Ultrasound

**DOI:** 10.3390/s26030884

**Published:** 2026-01-29

**Authors:** Dijian Xu, Yao Huang, Apurba Deb Mitra, Simon X. Yang, Ping Li, Mengqiu Xiao, Longbo Su, Lepeng Song

**Affiliations:** 1 School of Electronic and Electrical Engineering, Chongqing University of Science & Technology, Chongqing 401331, China; 2007050@cqust.edu.cn (D.X.); 2025205015@cqust.edu.cn (Y.H.); 2024204037@cqust.edu.cn (M.X.); 2023204006@cqust.edu.cn (L.S.); 2 State Key Laboratory of Power Transmission Equipment Technology, School of Electrical Engineering, Chongqing University, Chongqing 400044, China; apurbadebmitra@stu.cqu.edu.cn; 3 Advanced Robotics and Intelligent Systems Laboratory, School of Engineering, University of Guelph, Guelph, ON N1G 2W1, Canada; syang@uoguelph.ca; 4 Chongqing Academy of Agricultural Sciences, Chongqing 400039, China; liping@cqaas.cn

**Keywords:** switchgear, latency estimation, partial discharge, multiple local discharge power positioning

## Abstract

At present, most of the switchgear partial discharge detection means are offline detection and cannot monitor multiple partial discharge sources online at the same time. Based on this, this paper investigates the application of ultrasonic technology in localized discharge fault localization in high-voltage switchgear, removes the background noise of localized discharge in switchgear by using soft and hard filtering; proposes a generalized cubic correlation algorithm on the basis of TODA, improves the accuracy of the time difference acquisition in the case of low signal-to-noise ratio; determines the number of multiple localized discharging power sources by using the single-channel signal blind source separation technique and singularity spectral analysis; and determines the number of multiple localized discharging power sources by using independent component analysis to separate them. As well as for the problem that TDOA cannot be directly applied to the localization of multiple partial discharge sources, independent component analysis is used to separate the mixed signals, and the disordered coordinate selection method is proposed to determine the coordinates of multiple partial discharge sources. The experimental results show that (1) the noise reduction method is able to remove the excess interference while preserving the localized discharge signals; (2) the improved generalized cubic inter-correlation algorithm is more resistant to interference and has less error than other time delay estimation algorithms. The localization error is reduced by 60 mm~68 mm compared to the basic correlation algorithm, 41 mm~47 mm compared to the twice correlation algorithm, and 17 mm~20 mm compared to the three times correlation algorithm, which is a big improvement compared to the pre-improved algorithm. (3) It is able to locate the multiple localized power sources, and the accuracy of the number of localized power sources reaches 88%.

## 1. Introduction

With the continuous growth of China’s economy and the steady expansion of industrial production, the demand for electric power across various sectors has been rising sharply. As a result, national power grid companies have increasingly raised their standards and requirements for power supply services [1]. In the power system, high-voltage switchgear—widely deployed and present in large quantities—serves as an indispensable component of distribution networks [2], fulfilling critical functions such as control, protection, and switching of electrical energy. However, when insulation defects arise inside the switchgear, they cause an uneven electric field distribution, leading to a significant increase in the field strength at the defect location and ultimately inducing partial discharge. If such insulation defects are not detected and addressed in a timely manner, the resulting partial discharge may further develop into insulation breakdown of internal electrical components, causing a complete loss of insulation integrity [3]. It is noteworthy that partial discharge (PD) within switchgear typically occurs at atmospheric pressure, a process involving complex plasma dynamics. Modern research on atmospheric pressure discharges indicates that the discharge process often exhibits critical characteristics associated with the transition from glow or streamer discharge to high-energy arc discharge [4]. Specifically, this Glow-to-Arc Transition is generally driven by thermal instability, which induces rapid constriction of the discharge channel and the release of significant heat and pressure, thereby causing irreversible equipment damage [5]. Consequently, pinpointing the PD source using high-precision localization techniques during the incipient stages (e.g., glow or weak streamer phases) is of paramount physical significance for arresting fault evolution. Furthermore, Corona Discharge and Surface Dielectric Barrier Discharge represent the two most prevalent forms of early-stage faults within the insulation structures of switchgear. Recent studies indicate that corona discharge typically occurs in regions of high electric field concentration, such as electrode tips, and is accompanied by distinct acoustic pulse signatures [6]; conversely, surface discharge involves charge accumulation at the gas–solid interface and surface flashover mechanisms, presenting physical morphologies and discharge patterns that are significantly more complex than those of internal void discharges [7]. A comprehensive understanding of the physical mechanisms underlying these specific discharge types provides the theoretical foundation for the targeted ultrasonic localization algorithms proposed in this paper. Therefore, partial discharge within switchgear serves as a crucial indicator of the insulation condition of electrical equipment, and its effective detection is essential for ensuring the safe and stable operation of the power grid.

Partial discharge involves the generation of multiple physical phenomena, including heat, light, and sound; therefore, the presence of partial discharge inside a switchgear can be determined by detecting these phenomena [8]. At present, domestic and international detection methods for partial discharge faults in high-voltage switchgear typically employ infrared thermography, pulse current detection, radio-frequency detection, ultra-high-frequency detection (UHF), transient earth voltage detection (TEV), and ultrasonic detection, either individually or in combination [9]. Among these approaches, the ultra-high-frequency detection method (UHF) detects partial discharge (PD) by capturing the electromagnetic waves radiated during discharge events, offering excellent immunity to interference and thus being widely used for high-sensitivity monitoring inside equipment such as gas-insulated switchgear (GIS) [10]. The transient earth voltage detection method (TEV) exploits the voltage signals induced on the metallic enclosure by PD activity, making it suitable for convenient, non-intrusive patrol inspection of switchgear [11]. Owing to its outstanding insulation performance, the EFPI ultrasonic sensor exhibits promising application potential for anti-electromagnetic-interference detection [12]. Different detection modalities rely on distinct physical mechanisms and are therefore often complementary in practical applications. Given the complexity of the electromagnetic environment in on-site switchgear operation, ultrasonic detection based on acoustic principles has become an important diagnostic tool, as it is inherently immune to electromagnetic interference and facilitates geometric localization of discharge sources. To further improve the intelligence level and localization accuracy of PD detection, related research has been continuously advancing. In fault identification, convolutional neural network (CNN)-based methods have achieved high-accuracy defect-type recognition on specific datasets [13]. Wavelet-transform-based signal processing techniques have effectively improved the signal-to-noise ratio of noisy signals [14]. Mathematical morphology-assisted sparse-representation classifiers provide a theoretical basis for distinguishing single-point from multi-point PD sources [15]. Regarding localization technologies, the time-reversal method leverages electromagnetic-wave propagation characteristics and has demonstrated excellent performance in GIS localization [16]. The introduction of distributed feedback fiber laser (DFB-FL) arrays [17] and Mach–Zehnder interferometer fiber-optic sensors [18] has enriched the sensor options for multi-point detection and regional localization; moreover, integrated applications of UHF and optical sensors have further explored the potential of multidimensional information fusion [19]. For signal processing in complex field environments, researchers have proposed PRPD-based filtering algorithms [20] and UHF-synchronized ultrasonic denoising methods [21], aiming to enhance the extraction capability of weak signals. In addition, ultraviolet pulse detection systems, as an intuitive detection modality, possess unique practical value when discharge sites are visible [22]. In recent years, to address detection challenges under non-ideal conditions, noise separation and discriminative feature learning algorithms [23], improved RSS algorithms [24], and improved mantis search algorithms [25] have been proposed to strengthen system robustness in complex acoustic scenarios and under small-sample conditions. IoT-based microclimate monitoring architectures [26], distributed non-contact TEV systems [27], and field-reconstruction-based localization methods [28] also provide diversified technical pathways toward comprehensive perception and intelligent operation and maintenance of power equipment.

To address the aforementioned issues, building upon the ultrasonic detection methodology and specifically targeting low Signal-to-Noise Ratio (SNR) and multi-source mixing scenarios, this paper proposes a comprehensive suite of improved algorithms and a system integration scheme. The specific contributions and work are outlined as follows: First, a hardware-software collaborative filtering pipeline was constructed. This involves utilizing hardware filtering circuits for preliminary preprocessing of raw signals to eliminate interference components that deviate significantly from the central frequency of the partial discharge. Second, a high-order FIR band-pass filter was designed to perform secondary filtering on the partial discharge signals. This process employs the Fourier Transform to excise narrowband periodic interference, followed by a Wavelet Transform algorithm to suppress broadband white noise, thereby establishing a high-quality signal preprocessing front-end. Regarding Time Delay Estimation (TDE), an improved Generalized Triple Cross-Correlation (GTCC) algorithm is proposed. Tailored to the non-Gaussian characteristics of partial discharge signals, a novel weighting function based on the SNR maximization criterion was designed. This significantly enhances the estimation accuracy of the Time Difference of Arrival (TDOA) in low SNR environments. In terms of multi-source localization adaptation, a multi-source localization strategy based on Single-Channel Blind Source Separation (SCBSS) and Disordered Coordinate Selection was designed. This effectively resolves the challenge wherein traditional TDOA methods cannot be directly applied to multi-point discharges and scenarios with sequence ambiguity. Finally, precise localization of multi-source partial discharges was achieved utilizing a seven-element T-shaped localization model, verifying the effectiveness of the system in complex environments.

## 2. Background Noise Suppression for Partial Discharge

The essence of ultrasound-based partial discharge detection technology for switchgears lies in using ultrasonic sensors to receive partial discharge signals and subsequently analyze and locate them. The accuracy of ultrasonic localization is highly influenced by noise contained in the ultrasonic signals. Therefore, this chapter focuses on designing corresponding hardware and software filters based on the types and characteristics of interference in order to denoise the ultrasonic signals and lay the foundation for subsequent partial discharge localization.

### 2.1. Hardware Filtering

The operating environment of switchgears is highly complex, and various types of noise can significantly interfere with the acquisition of ultrasonic partial discharge signals. The center frequency of partial discharge is approximately 40 kHz. For interference components whose frequencies differ greatly from the PD center frequency (such as mechanical interference or corona pulse interference), analog filters can be used to suppress these disturbances.

**Figure 1:**
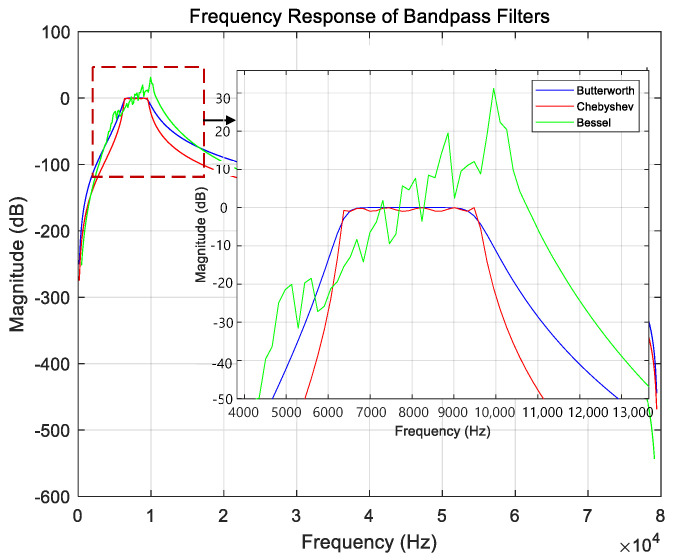
Common Filter Characteristics.

The commonly used filters are shown in Figure 1.

Based on the characteristics of the three filters shown in Figure 1, the Butterworth filter exhibits a flatter passband and more stable transition-band behavior compared with the Chebyshev and Bessel filters. Therefore, it is selected in this study for designing the band-pass filter. Since the filter order affects the attenuation rate in the transition band, and considering that low-frequency interference is stronger than high-frequency interference in the partial discharge environment of switchgears, a fourth-order high-pass filter and a second-order low-pass filter are cascaded to construct a band-pass filter. This configuration effectively suppresses low-frequency interference and enhances the performance of signal processing.

### 2.2. Filter Performance Simulation

**Figure 2:**
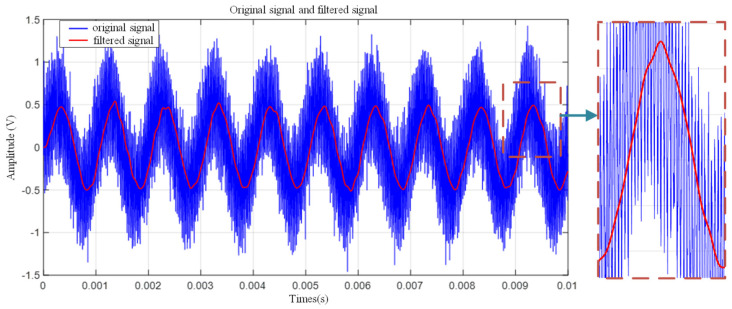
Filter simulation results.

To verify the frequency selection performance of the designed filter under strong interference environments, strict test conditions were set. Considering that high-amplitude sudden electromagnetic interference or mechanical noise may exist in the actual field, the amplitudes of four signal sources with different frequencies (40 kHz, 1 kHz, 1 MHz and white noise) were uniformly set to 0.5 V (which is approximately 50% of the peak value of a typical partial discharge signal). This was performed to test the filter’s ability to suppress strong out-of-band interference. As shown in Figure 2.

### 2.3. Digital Filtering

**Figure 3:**
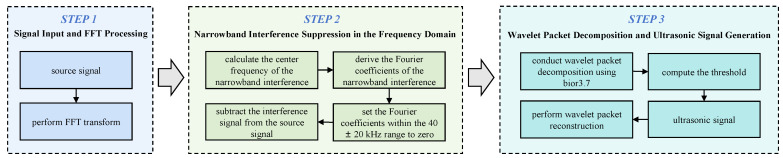
Digital Filtering Flowchart.

Due to the wide variety of components inside the switchgear, the interference signals generated during their operation are complex, resulting in severe interference in the field of partial discharge monitoring. Relying solely on hardware band-pass filters is no longer sufficient. Moreover, hardware band-pass filters can only perform coarse filtering over specific frequency bands, and their order cannot be designed too high due to self-oscillation and other factors. Electromagnetic coupling interference may also be present within the filter passband. In addition, white noise can affect the detection results. Therefore, digital denoising techniques are also required to further enhance the accuracy and reliability of signal processing. In this study, a finite impulse response (FIR) filter meeting specific performance requirements was designed with the assistance of MATLAB R2023a’s Filter Design Toolbox and fdatool. The filter has a passband of 30–100 kHz, transition bands of 20–30 kHz and 100–150 kHz, and a stopband attenuation of no less than 30 dB. When a function satisfies the Dirichlet conditions, it can be expanded using a Fourier series. The digital filtering workflow is shown in Figure 3.

It is important to clarify that the algorithm only sets the Fourier coefficients of the identified high-amplitude narrowband interference frequencies (e.g., the 60 kHz component) to zero, rather than suppressing the entire frequency band. Since the partial discharge signal is broadband, excising such extremely narrow interference bands results in negligible energy loss and minimal waveform distortion (specifically to the rising edge), thus ensuring the accuracy of the subsequent TDOA estimation.

### 2.4. Simulation Results of Digital Filtering

Partial discharge ultrasonic signals are characterized by a rapid rise followed by oscillatory decay. Therefore, a single-exponential decay function is employed to model the partial discharge ultrasonic signal $S_{d}$:

(1) $$S_{d}=\left\{\begin{array}{l} Ve^{\frac{-|t-t_{0}|}{\tau }}\cos\left(2\pi f_{0}t\right),\;t>t_{0}\\ 0,\;0\leq t<t_{0} \end{array}\right.$$

In the above equation, *V* represents the pulse amplitude, set to 1 V; *t*_0_ denotes the time at which the pulse reaches its peak; τ is the decay coefficient, set to 10; and $f_{0}$ represents the oscillation frequency, set to 40 kHz. Narrowband interference can be regarded as consisting of cosine or sine waves of different frequencies, each with varying amplitudes and phases.

**Figure 4:**
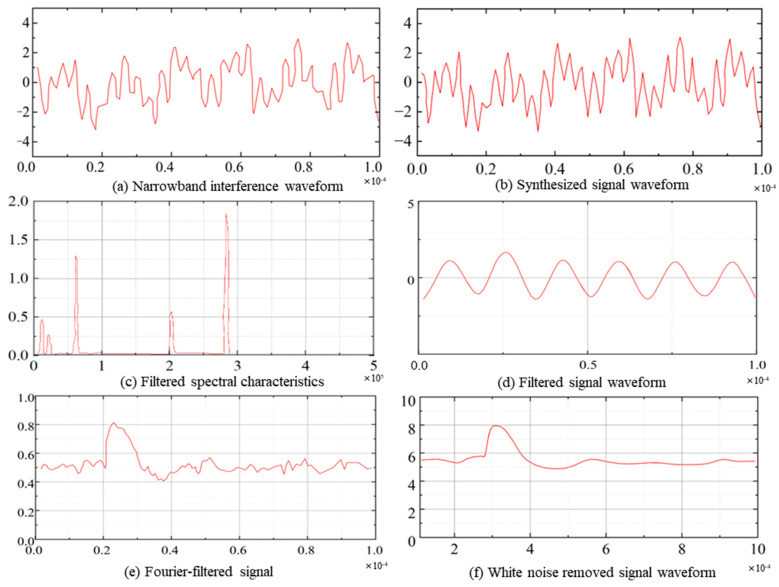
Digital Filter Simulation.

Cosine signals with frequencies of 10 kHz, 20 kHz, 60 kHz, 200 kHz, and 360 kHz were generated and superimposed (Figure 4a), clearly showing pronounced narrowband interference. After applying FIR filtering (Figure 4b), it was observed that the 60 kHz interference remained because it was still within the passband. Therefore, a Fourier-based algorithm was further employed to remove the remaining interference (Figure 4c), rendering the partial discharge signal more clearly visible in the frequency spectrum. Although the interference signals were attenuated, white noise effects persisted, necessitating denoising using wavelet packet decomposition (Figure 4d). Commonly used wavelet bases, including sym6, bior3.7, and rbio3.9, were screened, and the average entropy of their decomposition results was calculated (specific data are shown in Table 1, with results presented in (Figure 4e)).

The bior3.7 wavelet was ultimately selected as the optimal wavelet basis. As shown by the simulation results (Figure 4f), both narrowband interference and white noise were effectively suppressed, thereby enhancing the visibility of the partial discharge signal characteristics.

After a series of digital denoising processes, it can be observed that both narrowband interference and white noise have been effectively removed.

## 3. Time-Delay Estimation Algorithm

In TDOA algorithms, cross-correlation can be used to calculate the time difference of arrival between signals received by different sensors, where the peak of the correlation function corresponds to the desired time delay. However, basic cross-correlation introduces considerable errors under low signal-to-noise ratio conditions. To improve accuracy, second-order cross-correlation incorporates both autocorrelation and cross-correlation on the basis of basic cross-correlation. Generalized cross-correlation further adds a weighting function to enhance the sharpness of the correlation peak. Nevertheless, the operating environment of switchgear is complex, and various interferences pose challenges to acquiring partial discharge ultrasonic signals. Therefore, this study performs an additional round of correlation based on second-order cross-correlation and introduces a new weighting function, thereby forming an improved generalized third-order cross-correlation algorithm. As a result, the improved algorithm maintains distinct and sharp correlation peaks even under low SNR conditions.

Since third-order correlation involves multiple correlation operations, a large computational load is unavoidable. To address this issue, the improved algorithm is equipped with a fast Fourier transform (FFT)–based acceleration scheme. This enhanced time-delay estimation method significantly improves noise resistance and yields more accurate time-delay estimation results.

### 3.1. Fast Fourier Transform

In the context of signal correlation analysis, directly calculating higher-order correlation functions in the time domain imposes a substantial computational burden. Consequently, this study employs the Fast Fourier Transform (FFT) algorithm to transform discrete digital signals into the frequency domain for processing. According to the Convolution Theorem, the cross-correlation operation in the time domain is equivalent to the conjugate product of the signal spectra in the frequency domain. This property facilitates the rapid computation of the third-order cross-correlation function via frequency-domain multiplication. For the third-order cross-correlation $R_{RRR}(\tau )$, its corresponding frequency-domain representation (i.e., the third-order cross-energy spectrum $W_{RRR}(j\omega )$) can be directly expressed as the product of the individual signal spectral components:

(2) $$W_{RRR}\left(j\omega \right)=G_{G_{ss}^{*3}}\left(j\omega \right)\cdot e^{-j\omega \omega }+\sum_{i=1}^{N}G_{G_{u_{i}u_{i}}^{*3}}\left(j\omega \right)\cdot e^{-j\omega \omega_{\tau i}}+G_{G_{VV}^{*3}}\left(j\omega \right)\cdot e^{-j\omega \omega_{\tau V}}$$

where G(jω) denotes the spectrum of the respective signal components. By applying the Inverse Fast Fourier Transform (IFFT) to the above equation, the third-order cross-correlation function in the time domain is reconstructed. This approach significantly reduces the time complexity of the algorithm.

### 3.2. Generalized Cross-Correlation Time Delay Estimation Based on Third-Order Correlation Improvement

Traditional Generalized Cross-Correlation (GCC) algorithms primarily rely on second-order statistics. While effective under general noise conditions, their performance is often compromised when processing non-Gaussian Partial Discharge (PD) signals within switchgear characterized by strong Gaussian background noise. Given the inherent suppression property of Higher-Order Statistics (HOS) against Gaussian noise—specifically, the third-order cumulant of a Gaussian signal is theoretically zero—this paper introduces Cubic Correlation to enhance noise immunity. Furthermore, to render the peak of the correlation function sharper and more distinct, we designed a novel weighting function. This function integrates the characteristics of cubic correlation with the Generalized Cross-Correlation Time Delay Estimation (GCC-TDE) method based on Eckart weighting. This approach aims to minimize time delay estimation errors in low Signal-to-Noise Ratio (SNR) environments. Based on the preceding analysis:

(3) $$\begin{array}{l} W_{RRR}\left(j\omega \right)=F\left[R_{RRR}\left(\tau \right)\right]=G_{G_{ss}^{*3}}\left(j\omega \right)\cdot e^{-j\omega \omega }\\ +\sum_{i=1}^{N}G_{G_{u_{i}u_{i}}^{*3}}\left(j\omega \right)\cdot e^{-j\omega \omega_{\tau i}}+G_{G_{VV}^{*3}}\left(j\omega \right)\cdot e^{-j\omega \omega_{\tau V}} \end{array}$$

the newly designed weighting function is:

(4) $$H\left(j\omega \right)=\frac{aG_{G_{ss}^{*3}}}{{\left(\sum_{i=1}^{N}G_{G_{u_{i}u_{i}}^{*3}}\cdot G_{G_{VV}^{*3}}\right)}^{2}}$$

The weighting function H(jω) proposed in Equation (4) is formulated based on the Signal-to-Noise Ratio (SNR) maximization criterion. According to Higher-Order Statistics (HOS) theory, the third-order cumulant spectrum (Bispectrum) of Gaussian colored noise is theoretically zero. In contrast, Partial Discharge (PD) signals, being non-Gaussian impulsive signals, exhibit a third-order spectrum $G_{G_{ss}^{*3}}$ that contains significant characteristic information. The physical interpretation of this weighting function is analogous to that of the Eckart filter in Generalized Cross-Correlation (GCC): the numerator corresponds to the third-order spectral energy of the signal, while the denominator corresponds to that of the noise interference. Through this weighting mechanism, the algorithm automatically suppresses frequency bands with low SNR—dominated by $G_{G_{u_{i}u_{i}}^{*3}}$ and $G_{G_{VV}^{*3}}$—while enhancing those frequency bands containing the dominant energy of the PD signal. Although the form of this function serves as a heuristic extension based on Eckart weighting, it significantly improves the sharpness of the time delay estimation peak in scenarios where non-Gaussian signals are contaminated by Gaussian noise.

From observation of Equation (4), it is evident that this function significantly increases the weight of the effective signal while reducing the weight of noise signals when the signal-to-noise ratio decreases. When transformed back to the time domain, it emphasizes the time delay points, thereby markedly enhancing the algorithm’s robustness against noise interference. The detailed derivation of the generalized cross-correlation time delay estimation formula, improved via triple correlation, is provided in Appendix A.

### 3.3. Comparison and Analysis of Noise Immunity

Given that the primary research objective of this study is to enhance the Time Delay Estimation (TDE) accuracy of ultrasonic signals, the experimental validation focuses on a comparative analysis against classic algorithms within the same category, such as Cross-Correlation (CC) and Generalized Cross-Correlation with Phase Transform (GCC-PHAT). Since Ultra-High Frequency (UHF) and Transient Earth Voltage (TEV) detection methods rely on electromagnetic physical mechanisms, their data modalities are fundamentally distinct from acoustic signals. Consequently, they were not included in the quantitative comparison within the same dataset.

#### 3.3.1. Signal Collection

**Figure 5:**
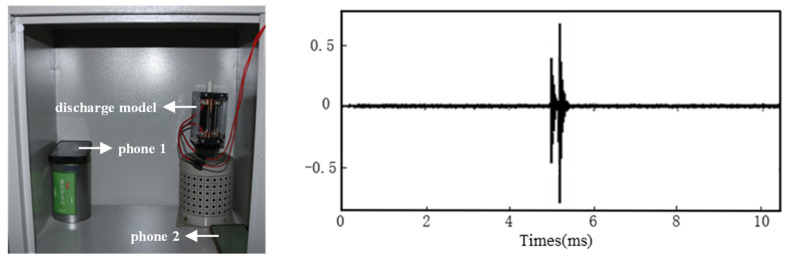
Noise resistance verification.

To evaluate the performance of the time delay estimation algorithm in a real noise environment, a physical experiment was conducted in this section. The experimental signals were derived from actual partial discharge ultrasonic waves generated by a needle-plate discharge model, with their spectral characteristics shown in Figure 5. Unlike the numerical simulation in Section 2.4, the signals collected here are real physical signals. To quantitatively test the algorithm’s noise immunity, Gaussian white noise of different powers was artificially superimposed on the collected raw signals to construct a series of test signals with known Signal-to-Noise Ratios (SNR).

In the multi-channel data acquisition setup, precise time synchronization is critical. Two smartphones linked via Bluetooth were utilized solely as a wireless synchronization trigger source. They generated simultaneous pulses that were recorded by the data acquisition system to establish a common time reference (zero-time alignment) for all channels during post-processing. This procedure allowed for the calibration and elimination of any fixed time delays between channels, thereby ensuring the accuracy of the subsequent time delay estimation.

#### 3.3.2. Noise Resistance Comparison

After completing the signal acquisition, random Gaussian white noise was added to each of the two signals, assuming that the Gaussian noise is independent of each other and also independent of the original ultrasonic signals. As mentioned above, it is assumed that under the same environmental conditions, the signal-to-noise ratio of the two signals remains consistent.

Using a needle-plate discharge model to simulate partial discharge ultrasonic signals, experiments were conducted under different signal-to-noise ratios employing basic cross-correlation, second-order correlation, third-order correlation, and generalized third-order cross-correlation. These experiments were designed to verify the accuracy and superiority of the proposed improved algorithm.

**Figure 6:**
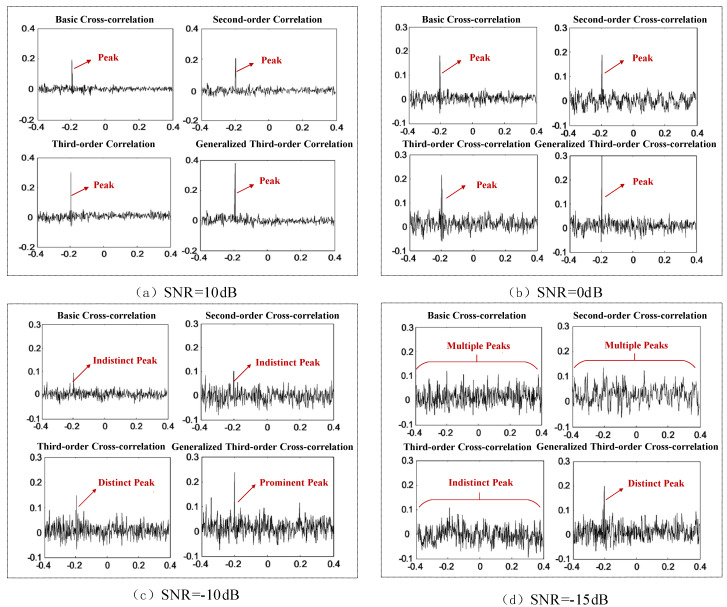
Comparison of correlation functions of the four algorithms under different SNR conditions.

When the signal-to-noise ratio is relatively high (10 dB) (Figure 6a), the correlation peaks of all four algorithms maintain good resolution, with negligible differences in error. As the signal-to-noise ratio decreases to 0 dB (Figure 6b), although the noise level increases, the correlation peaks of all four algorithms remain relatively distinct. When the signal-to-noise ratio further drops to −10 dB (Figure 6c), external interference becomes significant, and the peak values of the basic cross-correlation and second-order correlation algorithms become noticeably unstable, with many spurious peaks appearing. At a signal-to-noise ratio of −15 dB (Figure 6d), interference is even stronger, and multiple peaks emerge in the basic, second-order, and third-order correlation results, making accurate peak identification difficult. In contrast, the improved generalized third-order correlation algorithm still maintains relatively sharp correlation peaks at −15 dB, demonstrating superior noise robustness.

## 4. Localization of Multiple Partial Discharge Sources in Switchgear

Due to the complex operating environment of switchgear and the diversity of its internal components, multiple partial discharge sources can often occur simultaneously within the switchgear. To address this issue, the following approach is proposed: (1) A single-channel signal blind source separation technique combined with singular spectrum analysis is employed to determine the number of partial discharge sources, and virtual multi-channel signals are constructed using principal component analysis to separate the mixed ultrasonic signals. (2) Multi-source partial discharge localization is achieved using a disorderly coordinate selection method together with a seven-element T-shaped positioning model, effectively addressing the issue that mixed signals from multiple partial discharge sources cannot be directly applied to TDOA-based localization.

### 4.1. Single-Channel Signal Blind Source Separation

In the localization of multiple partial discharge sources, the information content provided by the source signals generated by the partial discharge sources is relatively low, unlike the original signals. The original signals can be understood as the ultrasonic signals produced by the partial discharge sources, which reach the sensors after propagation, time delays, and other effects, but without undergoing any fusion. Ultrasonic sensors perform fusion on multiple original signals and thus can be regarded as an instantaneous mixing device that linearly superimposes the signals. During single-channel signal blind source separation, the original signals can be treated as the source signals, and the signal mixing model can be regarded as an instantaneous mixing model.

**Figure 7:**
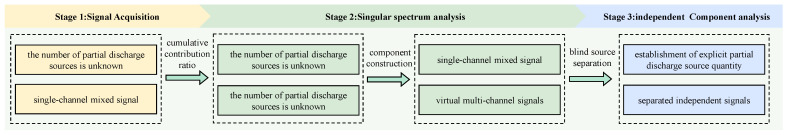
A Hybrid Signal Separation Framework Based on Single Channel Signal Blind Source Separation.

Based on the above analysis, the flowchart of the single-channel signal blind source separation technique is shown in Figure 7.

### 4.2. Determination of the Number of Multiple Partial Discharge Sources

The core principle of employing Singular Spectrum Analysis (SSA) to identify the number of multiple partial discharge sources within switchgear involves mapping a one-dimensional time series into a multi-dimensional space. Separation is achieved by exploiting the energy disparity between the signal and noise within the feature subspace. The primary steps are as follows:

1. Construction of Trajectory Matrix and Eigen Decomposition

The acquired one-dimensional ultrasonic signal data of length l is transformed into a trajectory matrix Y. The construction of the trajectory matrix utilizes time-delayed copies of the signal, enabling effective extraction of the dynamical structure of the time series. Performing eigenvalue decomposition on the matrix $Y^{T}Y$ yields a series of eigenvalues arranged in descending order: $\lambda_{1},\lambda_{2},\ldots ,\lambda_{m}$. According to Singular Value Decomposition (SVD) theory, larger eigenvalues correspond to the dominant energy components of the signal (i.e., the partial discharge sources), whereas smaller eigenvalues typically correspond to noise interference.

2. Calculation of Cumulative Contribution Rate

To quantitatively assess the importance of each component, the contribution rate $CR_{i}$ of the i-th eigenvalue and the cumulative contribution rate $CCR_{k}$ of the first k eigenvalues are calculated as:

(5) $$CR_{i}=\frac{\lambda_{i}}{\sum_{j=1}^{m}\lambda_{j}}$$

(6) $$CCR_{k}=\frac{\sum_{i=1}^{k}\lambda_{i}}{\sum_{j=1}^{m}\lambda_{j}}$$

3. Identification of the Number of Partial Discharge Sources

Since the actual signal is composed of the superposition of N partial discharge source signals and background noise, this manifests in the feature space as N+1 principal components. An energy threshold β is established (set to 0.85 in this paper). When the cumulative contribution rate $CCR_{\left(N+1\right)}$ exceeds this threshold for the first time, the count of independent components is determined to be N+1, thereby identifying the number of partial discharge sources as N.

### 4.3. Separation of Mixed Signals

#### 4.3.1. Construction of Virtual Multi-Channels

1. Select a new window length to construct the trajectory matrix $Y_{new}$, and compute *M* eigenvectors $q_{1}$, $q_{2}$, …, $q_{M}$.

2. Construct the subspace matrices. Build *M* subspace matrices, where the *i*-th subspace matrix is defined as:

(7) $$Y_{i}^{\prime }=Y_{new}q_{i}q_{i}^{T}$$

In this expression, $q_{i}q_{i}^{T}$ forms the *i*-th component of $Y_{new}$, and projecting $Y_{0}$ onto this subspace yields $Y_{i}^{\prime }$.

3. Construct the component matrices. According to the number of partial discharge sources *N*, establish the corresponding *N* component matrices, where the *i*-th component matrix is given by:

(8) $$U_{i}=\left\{\begin{array}{l} Y_{i}^{\prime },\;i=1,2,\cdots ,N-1\\ \sum_{j=N}^{M}Y_{j}^{\prime },\;i=N \end{array}\right.$$

4. Construct the virtual multi-channel matrix. The elements on the *j*-th subdiagonal of the component matrix $U_{i}$ form a vector $D_{i,j}$ of length χ. Accordingly, the *j*-th element of the *i*-th virtual multi-channel vector is defined as:

(9) $$z_{i}\left(j\right)=\frac{\sum_{k=1}^{\chi }D_{i,j}\left(k\right)}{\chi }$$

This step performs diagonal averaging of the signal. The resulting virtual multi-channel matrix is thus obtained as:

(10) $$Z=\left[z_{1};z_{2};\cdots ;z_{N}\right]$$

The window length has various effects on singular spectrum analysis; in this work, the window length *M* is set to 20.

#### 4.3.2. Separation of Mixed Ultrasonic Signals

Regarding the applicability of the ICA algorithm within the switchgear acoustic environment, this paper formulates assumptions based on the following physical and statistical characteristics: (1) Non-Gaussianity: Partial discharge (PD) ultrasonic signals manifest as transient pulses with steep wavefronts. Their statistical distribution exhibits significant sparsity and high excess kurtosis, strictly satisfying the non-Gaussianity requirement of ICA for source signals and distinguishing them fundamentally from Gaussian-distributed background noise. (2) Statistical Independence and De-reverberation: Although the multipath effect and reverberation within the switchgear introduce time-delayed correlations, the physical generation mechanisms of distinct partial discharge sources (e.g., corona versus surface discharge) possess microscopic stochasticity. Consequently, their pulse sequences are statistically independent in their temporal distribution. Furthermore, the Singular Spectrum Analysis (SSA) step introduced in this paper embeds the single-channel signal into a high-dimensional phase space by constructing a trajectory matrix. This process leverages the temporal structure of the signal to preliminarily separate the primary source signal components from the complex, low-energy reverberation components within the subspace. This enables the subsequent ICA algorithm to effectively extract statistically independent principal component signals from the virtual multi-channels.

Independent Component Analysis (ICA) is a technique for separating instantaneous mixed signals. It relies on three conditions: statistical independence of the source signals, at most one source signal following a Gaussian distribution, and the number of observed signals being no less than the number of source signals. Under these assumptions, the observed signals can be decomposed into a linear combination of non-Gaussian sources. During ICA processing, the original data set is first centered to remove the mean, followed by whitening to obtain statistically independent signals with unit variance, producing matrix *V*. A target function is then constructed with respect to the separation matrix *W*. The Fast ICA algorithm adopted in this study is a well-established method in blind source separation. It iteratively maximizes the non-Gaussianity of the signal $W^{T}V$ to effectively separate the source components. The iterative update formula for computing the separation matrix *W* is:

(11) $$W_{k}=E\left[Vg\left(W_{k-1}^{T}V\right)\right]-E\left[g^{\prime }\left(W_{k-1}^{T}V\right)\right]W_{k-1}$$

It is worth noting that while ICA relies on the assumption of statistical independence, implies distinct physical behaviors. Even if two PD sources are synchronous on the macro-scale (power frequency cycle), they exhibit stochastic differences on the micro-scale (amplitude randomness, inception jitter). Furthermore, the Singular Spectrum Analysis (SSA) introduced in this paper (Section 4.3.1) decomposes the signal into orthogonal subspaces via SVD, effectively de-correlating the components before ICA processing. Combined with the spatial diversity of the mixing matrix (due to different propagation paths), the proposed SSA-ICA method retains separation capability for highly correlated sources, although we acknowledge in Section 6 that performance degrades compared to strictly independent sources.

### 4.4. Localization of Multiple Partial Discharge Sources

#### 4.4.1. Coordinate Selection Based on Unordered Grouping of Separated Signals

Following the processing of multi-source mixed signals using Blind Source Separation (BSS) techniques, the inherent permutation ambiguity of the algorithm disrupts the correspondence between the sequence of the separated signals and the physical locations of the source signals. Consequently, the extracted time differences cannot be directly substituted into the Time Difference of Arrival (TDOA) equation system for solution. To resolve this specific adaptation challenge and achieve the correct matching between separated signals and spatial coordinates, this paper proposes a Disordered Coordinate Selection Method. In three-dimensional space, localizing partial discharge sources using TDOA technology necessitates a minimum of four ultrasonic sensors. To facilitate analysis, we present eight configuration combinations involving four ultrasonic sensors and two partial discharge sources. Let $t_{i,j}$ denote the arrival time of the ultrasonic signal generated by the *j*-th partial discharge source at the *i*-th ultrasonic sensor, and let $PD_{k,j}$ denote the coordinates of the *j*-th partial discharge source obtained from the *k*-th configuration. The eight possible permutations of signal assignments and source coordinates are summarized in Table 2. 

#### 4.4.2. Positioning Model

**Figure 8:**
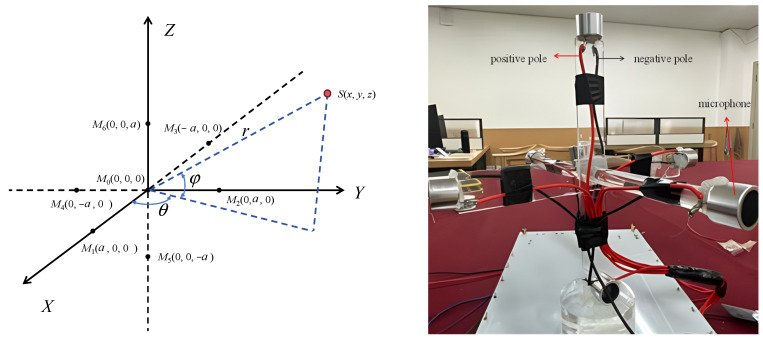
Seven element T-shaped positioning model.

The seven-element T-shaped localization model provides effective global positioning in three-dimensional space. Its placement within the coordinate system is shown in Figure 8.

The coordinates of the sensors in the coordinate system are as follows: *M*_0_(0, 0, 0), *M*_1_(*D*, 0, 0), *M*_2_(0, *D*, 0), *M*_3_(−*D*, 0, 0), *M*_4_(0, −*D*, 0), *M*_5_(0, 0, −*D*), and *M*_6_(0, 0, *D*). where *D* denotes the distance between the *i*-th sensor *M_i_* and the reference sensor *M_0_* located at the origin. The Cartesian coordinates of the partial discharge source are *s*(*x*, *y*, *z*), with azimuth angle *θ* and elevation angle *φ*. The distance from the source to the coordinate origin is *r*, and the distance difference from the source to the *i*-th microphone is *d_i_* 1 ≤ *i* ≤ 6. (In the figure, *M_0_* is the origin.) Let *τ_i_* represent the estimated time delay of the signal received between *M_0_* and *M_i_* 1 ≤ *i* ≤ 6.

Based on these conditions, the following relationships can be derived:

(12) $$\left\{\begin{array}{l} x^{2}+y^{2}+z^{2}=r^{2}\\ {\left(x-D\right)}^{2}+y^{2}+z^{2}={\left(r+d_{1}\right)}^{2}\\ x^{2}+{\left(y-D\right)}^{2}+z^{2}={\left(r+d_{2}\right)}^{2}\\ {\left(x+D\right)}^{2}+y^{2}+z^{2}={\left(r+d_{3}\right)}^{2}\\ x^{2}+{\left(y+D\right)}^{2}+z^{2}={\left(r+d_{4}\right)}^{2}\\ x^{2}+y^{2}+{\left(z+D\right)}^{2}={\left(r+d_{5}\right)}^{2}\\ x^{2}+y^{2}+{\left(z-D\right)}^{2}={\left(r+d_{6}\right)}^{2} \end{array}\right.$$

From Equation (12), it can be solved that:

(13) $$r=\frac{-6D^{2}+c^{2}\sum_{i=1}^{6}\tau_{i}^{2}}{2c\sum_{i=1}^{6}\tau_{i}}$$

(14) $$\tan\varphi =\frac{\left(\tau_{2}-\tau_{4}\right)\left[2r+c\left(\tau_{2}+\tau_{4}\right)\right]}{\left(\tau_{1}-\tau_{3}\right)\left[2r+c\left(\tau_{1}+\tau_{3}\right)\right]}$$

(15) $$\tan\theta =\frac{\sqrt{{\left(\left(\tau_{1}-\tau_{3}\right)\left[2r+c\left(\tau_{1}+\tau_{3}\right)\right]\right)}^{2}+{\left(\left(\tau_{2}-\tau_{4}\right)\left[2r+c\left(\tau_{2}+\tau_{4}\right)\right]\right)}^{2}}}{\left(\tau_{6}-\tau_{5}\right)\left[2r+c\left(\tau_{6}+\tau_{5}\right)\right]}$$

In the equation, *r* is the distance from the coordinate *s*(*x*, *y*, *z*) to the origin (0, 0, 0), φ is the angle between the line OS and its projection on the xy-plane, and θ the angle between this projection and the *X*-axis.

From Equations (13)–(15), r, φ and θ can be obtained, giving the spherical coordinates of the partial discharge source. Then, according to Equation (16):

(16) $$\left\{\begin{array}{l} x=r\cos\varphi \cos\theta \\ y=r\cos\varphi \sin\theta \\ z=r\sin\varphi \end{array}\right.$$

The spatial coordinates of the partial discharge source, *s*(*x*, *y*, *z*), can thus be determined.

## 5. Experimental Study on Multiple Partial Discharge Sources in Switchgear

### 5.1. Partial Discharge Localization Model

**Figure 9:**
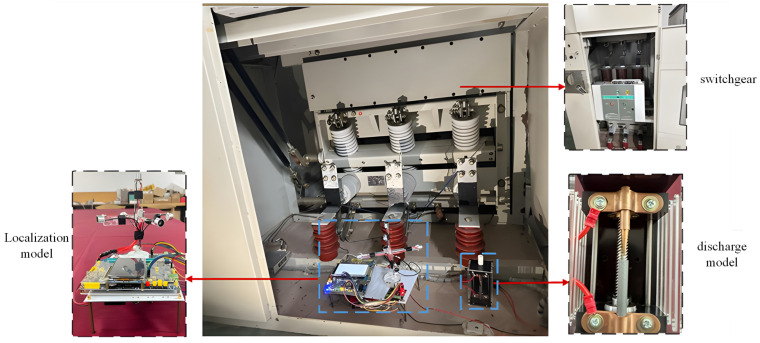
On-Site Experiment for Multiple Partial Discharge Sources in Switchgear.

The previously designed partial discharge model and localization model were connected, and after energizing the high-voltage system, the partial discharge fault model was subjected to step-up voltage treatment according to the 0.1 kV/s switchgear discharge model voltage rise plan. The discharge signals generated during each partial discharge event were recorded sequentially, converted from acoustic to electrical signals, and then saved and exported to provide data support for subsequent analysis. The on-site experimental setup is shown in Figure 9.

**Figure 10:**
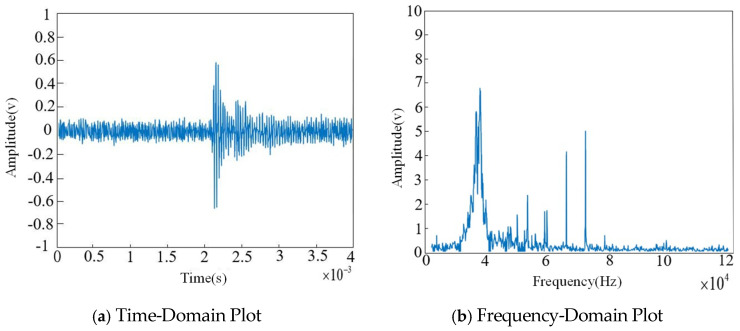
Ultrasonic signal diagram when corona discharge occurs in the switchgear.

Since corona discharge accounts for a significant proportion of partial discharge types in high-voltage switchgear and produces relatively large waveform amplitudes, the experimental analysis is conducted using a corona discharge model as an example. The time-domain and frequency-domain results of the corona discharge are shown in Figure 10.

As shown in the figure, due to the presence of noise, the ultrasonic signal exhibits many spikes (narrowband interference), and the original signal may be submerged within the white noise.

**Figure 11:**
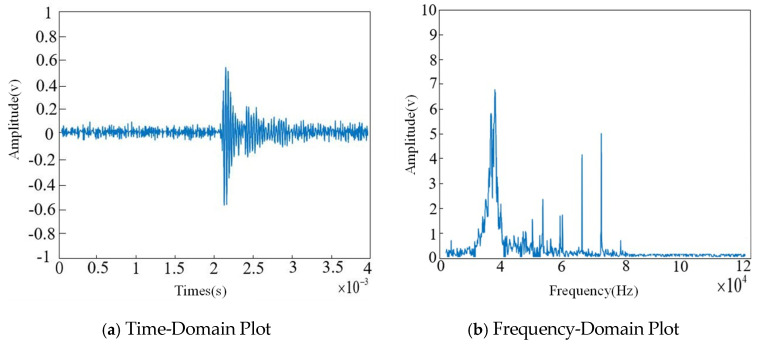
Signal waveform after FIR filtering.

The time-domain and frequency-domain plots after FIR filtering are shown in Figure 11.

**Figure 12:**
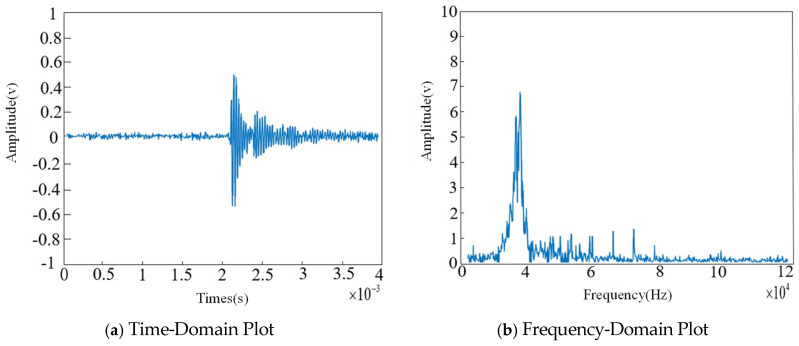
Fourier Transform for Narrowband Interference Removal.

As shown in the figure, while effectively preserving the useful signals generated during partial discharge events, the environmental noise present when no discharge occurs has been largely removed. However, narrowband interference and white noise still exist within the ultrasonic signals of the partial discharge, necessitating further removal of narrowband interference using the Fourier-based algorithm. The processing results are presented in Figure 12.

**Figure 13:**
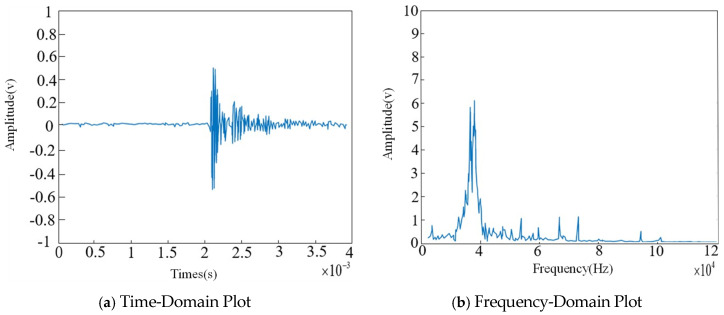
Wavelet Transform for Removing White Noise.

As shown in the figure, after processing with the Fourier-based algorithm, much of the narrowband interference has been removed; however, white noise may still be present in the ultrasonic signals. Therefore, wavelet packet transformation is applied to eliminate the white noise. The time-domain and frequency-domain results of this processing are shown in Figure 13.

As shown in the figure, after applying the wavelet packet transformation, the white noise has been effectively removed. In the time domain, the acquired ultrasonic signals retain the useful components while achieving a high degree of filtering of environmental noise, and the frequency bands of the useful signals remain largely unaffected. Therefore, the digital filtering method proposed in this study can effectively eliminate background noise.

### 5.2. Comparison and Analysis of Time Delay Estimation Errors

Given that the primary focus of this study is the improvement of Time Delay Estimation (TDE) accuracy for ultrasonic signals, our experimental framework was specifically designed to benchmark against classical cross-correlation algorithms within the same domain (e.g., CC, GCC-PHAT). As UHF and TEV detection methods are predicated on electromagnetic physical mechanisms, their data modalities differ fundamentally from acoustic signals. Consequently, they were not included in the quantitative comparison on the same dataset.

The Root Mean Square Error (RMSE) is a standard metric for evaluating the deviation between observed values and true values. By collecting data from multiple experiments, the results of four different time delay estimation methods—conventional cross-correlation, second-order correlation, third-order correlation, and generalized third-order cross-correlation—can be compared against the true values to more clearly demonstrate their respective performances. Furthermore, by varying the signal-to-noise ratio, different levels of noise interference can be simulated, allowing for the assessment of these methods’ robustness against noise. The RMSE of the time delay estimates is as follows:

(17) $$EMSE=\sqrt{\frac{\sum_{i=1}^{N}d_{\epsilon }^{2}}{N}}$$

In Equation (17), *d_i_* represents the deviation between the *i*-th measured value and the true value.

**Figure 14:**
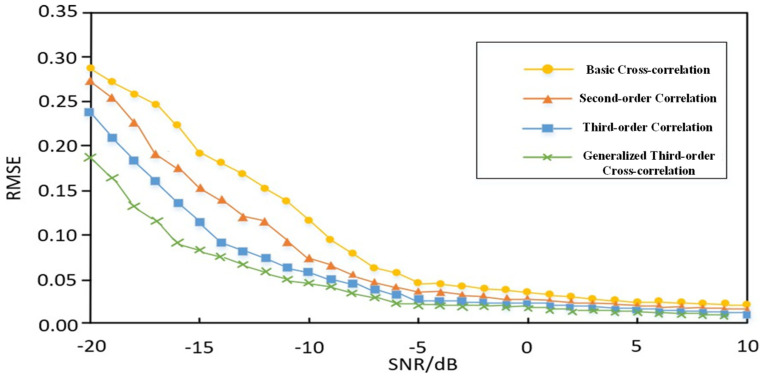
Comparison of Time Delay Estimation Performance of Four Related Algorithms.

Taking the case of two sensors and a single partial discharge source as an example, multiple experiments were conducted. For ultrasonic signals collected under different signal-to-noise ratio conditions, 100-time delay estimations were performed using the four aforementioned time delay estimation methods. The resulting estimation errors are shown in Figure 14. In the figure, the horizontal axis represents the signal-to-noise ratio, ranging from −20 dB to 10 dB, while the vertical axis represents the calculated root mean square error.

As shown in Figure 14, when the signal-to-noise ratio is relatively high, the errors of the four algorithms do not differ significantly. However, as the signal-to-noise ratio decreases, the generalized third-order cross-correlation algorithm demonstrates markedly superior anti-interference capability compared to the other three methods. Therefore, the generalized third-order cross-correlation time delay estimation algorithm is better suited for complex working environments with high levels of noise interference.

To further verify the performance of each algorithm, a localization experiment was conducted.

The coordinate of sensor 4, the central sensor of the localization model described in Section 4.4, was taken as the origin (0, 0, 0) mm. Given that the distance between each sensor is 80 mm, the coordinates of the remaining sensors are (80, 0, 0) mm, (0, 80, 0) mm, (−80, 0, 0) mm, (0, −80, 0) mm, (0, 0, −80) mm, and (0, 0, 80) mm. A partial discharge source was placed at coordinates (400, 400, 400) mm. Localization experiments were conducted using basic cross-correlation, second-order correlation, third-order correlation, and generalized third-order cross-correlation algorithms. The localization results are presented in Table 3, where a, b, c, and d correspond to the results obtained by the different time delay estimation algorithms for the corona discharge model, surface discharge model, air-gap discharge model, and floating discharge model, respectively.

As shown by the results in the table, the localization error using basic cross-correlation ranges from approximately 100 mm to 118 mm, while the error for second-order correlation is around 81 mm. Third-order correlation shows a significant improvement, with errors ranging from approximately 57 mm to 70 mm. The generalized third-order cross-correlation algorithm proposed in this study further reduces the error based on the third-order correlation, achieving an error range of approximately 40 mm to 50 mm. These experimental results demonstrate that the improved algorithm exhibits a substantial enhancement in performance.

### 5.3. Multi-Source Partial Discharge Localization Experiment

#### 5.3.1. Calculation of the Number of Multiple Partial Discharge Sources

During the construction of the trajectory matrix, selecting a small window length *m* can cause the cumulative contribution rate *CCR* to rise rapidly and approach 1, which may reduce its ability to distinguish between different trajectories. Conversely, choosing a large window length can cause the *CCR* to become overly dispersed, resulting in excessively sparse information in the matrix, which also affects its effectiveness. Based on a literature review and experimental trials, the window length *m* in this study is set to 50.

**Figure 15:**
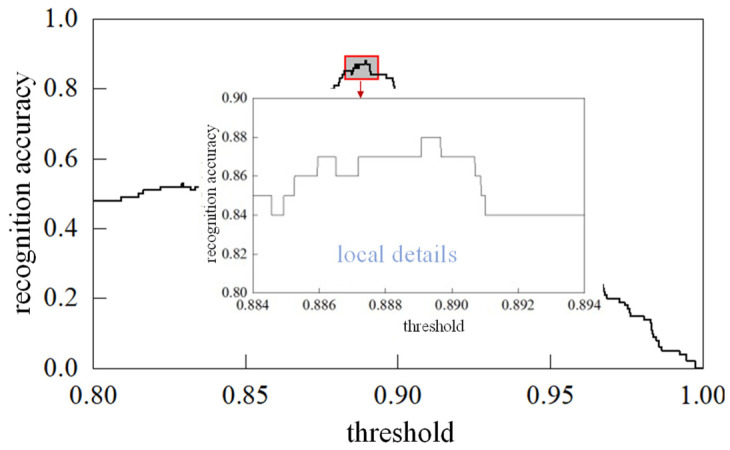
The relationship between threshold and identification of the number of partial discharge sources.

As described in Section 4.2, when identifying the number of partial discharge sources, the cumulative contribution rate is calculated, and a threshold *β* is set. If *CCR* (*N* + 1) first reaches or exceeds *β*, the number of partial discharge sources is determined to be *N*. To analyze the relationship between the threshold and the identification accuracy of the number of partial discharge sources, this study collected 50 sets of ultrasonic signals generated by a single partial discharge source and 50 sets generated by multiple partial discharge sources. The cumulative contribution rates of these signals were calculated, and an analysis was conducted to plot the relationship between the identification accuracy and different threshold values, as shown in Figure 15. When the threshold *β* is within the range [0.88916, 0.88976], the identification accuracy reaches a maximum of 0.88. Therefore, the threshold is set to *β* = 0.8895 in this study.

**Figure 16:**
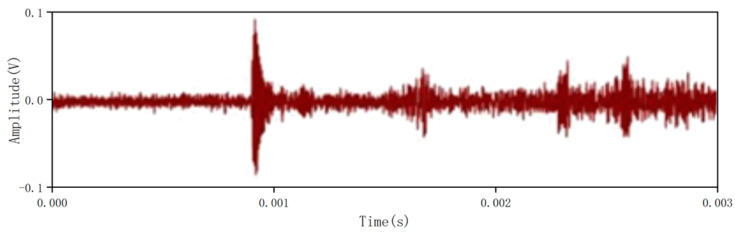
Ultrasonic signals generated by multiple local discharge sources received by sensors.

Taking the commonly encountered case of two partial discharge sources as an example, the corona discharge model and surface discharge model were used to generate controllable partial discharge signals. The signal received by a particular sensor is shown in Figure 16.

**Figure 17:**
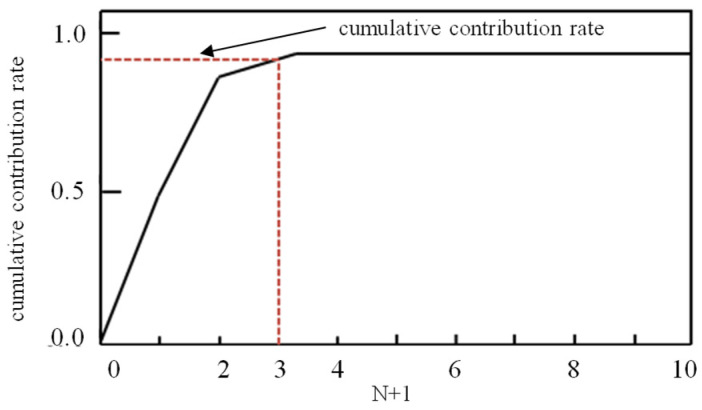
Estimation of the number of partial discharge sources.

Taking the mixed signal received by this sensor as an example, its cumulative contribution rate is shown in Figure 17. When *N* + 1 = 3, the cumulative contribution rate first exceeds 0.8895, indicating that the number of partial discharge sources is 3 − 1 = 2.

#### 5.3.2. Calculation of Coordinates for Multiple Partial Discharge Sources

Based on the mixed discharge scenario established in Section 5.3.1 (characterized by the simultaneous presence of corona and surface discharges), the proposed system utilizes Single-Channel Blind Source Separation (SCBSS) technology to overcome the aliasing interference of the two discharge signals in the time-frequency domain. The separated independent components correspond, respectively, to the corona and surface discharge signals, and their localization results are presented in Table 4.

Based on the table, it can be seen that when multiple partial discharge sources are present, the coordinate selection method proposed in this paper can effectively localize the coordinates of multiple partial discharge sources.

## 6. Conclusions

This study targets the engineering challenge of online localization of multiple partial discharge (PD) sources in switchgear and establishes a complete system pipeline of “hardware filtering–algorithmic denoising–blind source separation–time-delay-based localization”. The main contributions are as follows: (1) it is verified that the improved generalized third-order cross-correlation algorithm significantly enhances the accuracy of time-delay estimation under low SNR conditions (−15 dB), thereby overcoming the noise-robustness bottleneck of conventional second-order correlation methods; (2) it is demonstrated that combining single-channel blind source separation (SCBSS) with an unordered coordinate selection strategy effectively resolves the inversion problem of multi-source mixed signals, enabling an accurate mapping from mixed signals to the coordinates of independent acoustic sources; (3) experimental results show that the proposed method can achieve three-dimensional localization of more than two PD sources with an error below 50 mm in a complex electromagnetic environment, providing an effective acoustic solution for non-intrusive diagnosis of insulation defects in switchgear.

However, as an exploratory study, this work still has objective limitations in terms of physical boundaries and the scope of comparison: (1) constrained by the narrow space inside switchgear and the Rayleigh criterion, the 80 mm array aperture limits far-field angular resolution; when the source distance exceeds 2 m, the localization error increases nonlinearly with distance, restricting the coverage of single-station monitoring; (2) limited by experimental conditions, this study conducted algorithmic comparisons only within the ultrasonic modality; a synchronized multimodal dataset incorporating UHF and TEV was not established, and cross-modality, system-level performance benchmarking has not yet been performed; (3) although the current single-point processing latency of 0.8 s, together with an “event-triggered” mechanism, is sufficient for online diagnosis at the level of condition-based maintenance, the computational overhead of high-dimensional matrix operations makes it difficult to capture millisecond-scale transient evolution, and the statistical independence assumption of blind source separation still faces theoretical boundary challenges under extremely symmetric discharge scenarios.

To address these limitations and meet the requirements for engineering deployment, future work will be conducted along three dimensions: (1) optimizing operation-and-maintenance strategy and cost: to cope with large-scale deployment constraints, the system will be positioned as a mobile “high-precision diagnostic tool” to support hierarchical inspection with portable devices, and MEMS ultrasonic arrays will be introduced to replace piezoelectric sensors to improve economic feasibility; (2) hardware acceleration and improved real-time performance: leveraging GPU parallel computing and edge-AI chip technologies, we will develop an FPGA-based parallel acceleration architecture to pipeline the computationally intensive third-order correlation operations, thereby meeting real-time requirements in ultra-high-frequency discharge scenarios; (3) multimodal fusion and algorithm robustness: we will construct a joint acoustic–electrical monitoring dataset, investigate deep-clustering-based discharge pattern recognition to replace conventional statistical assumptions, and introduce dynamic aperture expansion techniques to enhance comprehensive far-field diagnostic capability.

## Figures and Tables

**Table 1:** Entropy value of wavelet packet decomposition based on different wavelet bases.

	First Time	Second Time	Third Time	Fourth Time	Average
sym6	4.6546	2.035	3.5329	2.0161	2.7208
bior3.7	18.635	0.9967	8.5492	−30.014	−0.4802
rbio3.9	4.5536	2.2772	3.1626	4.5802	3.7096

**Table 2:** Schematic diagram of signal arrival time and coordinate arrangement combination.

Index	TDOA Combination 1	Coordinate 1	TDOA Combination 1	Coordinate 2
1	*t*_2,1_ − *t*_1,1_, *t*_3,1_ − *t*_1,1_, *t*_4,1_ − *t*_1,1_	*PD*_1,1_	*t*_2,2_ − *t*_1,2_, *t*_3,2_ − *t*_1,2_, *t*_4,2_ − *t*_1,2_	*PD*_1,2_
2	*t*_2,1_ − *t*_1,1_, *t*_3,1_ − *t*_1,1_, *t*_4,2_ − *t*_1,1_	*PD*_2,1_	*t*_2,2_ − *t*_1,2_, *t*_3,2_ − *t*_1,2_, *t*_4,1_ − *t*_1,2_	*PD*_2,2_
3	*t*_2,1_ − *t*_1,1_, *t*_3,2_ − *t*_1,1_, *t*_4,1_ − *t*_1,1_	*PD*_3,1_	*t*_2,2_ − *t*_1,2_, *t*_3,1_ − *t*_1,2_, *t*_4,2_ − *t*_1,2_	*PD*_3,2_
4	*t*_2,1_ − *t*_1,1_, *t*_3,2_ − *t*_1,1_, *t*_4,2_ − *t*_1,1_	*PD*_4,1_	*t*_2,2_ − *t*_1,2_, *t*_3,1_ − *t*_1,2_, *t*_4,1_ − *t*_1,2_	*PD*_4,2_
5	*t*_2,2_ − *t*_1,1_, *t*_3,1_ − *t*_1,1_, *t*_4,1_ − *t*_1,1_	*PD*_5,1_	*t*_2,1_ − *t*_1,2_, *t*_3,2_ − *t*_1,2_, *t*_4,2_ − *t*_1,2_	*PD*_5,2_
6	*t*_2,2_ − *t*_1,1_, *t*_3,1_ − *t*_1,1_, *t*_4,2_ − *t*_1,1_	*PD*_6,1_	*t*_2,1_ − *t*_1,2_, *t*_3,2_ − *t*_1,2_, *t*_4,1_ − *t*_1,2_	*PD*_6,2_
7	*t*_2,2_ − *t*_1,1_, *t*_3,2_ − *t*_1,1_, *t*_4,1_ − *t*_1,1_	*PD*_7,1_	*t*_2,1_ − *t*_1,2_, *t*_3,1_ − *t*_1,2_, *t*_4,2_ − *t*_1,2_	*PD*_7,2_
8	*t*_2,2_ − *t*_1,1_, *t*_3,2_ − *t*_1,1_, *t*_4,2_ − *t*_1,1_	*PD*_8,1_	*t*_2,1_ − *t*_1,2_, *t*_3,1_ − *t*_1,2_, *t*_4,1_ − *t*_1,2_	*PD*_8,2_

**Table 3:** (**a**) Comparison of Corona Discharge Positioning Results. (**b**) Comparison of Surface Discharge Positioning Results. (**c**) Comparison of Air Gap Discharge Positioning Results. (**d**) Comparison of Suspension Discharge Positioning Results.

(a)				
Number of Experiments	Fundamental Cross-Correlation Error (*x*, *y*, *z*) mm	Quadratic Correlation Error (*x*, *y*, *z*) mm	Cubic Correlation Error (*x*, *y*, *z*) mm	Generalized Cubic Correlation Error (*x*, *y*, *z*) mm
1	(110.23, 110.63, 109.84)	(95.32, 93.02, 84.36)	(64.63, 69.37, 61.29)	(45.28, 48.36, 42.65)
2	(112.03, 118.62, 104.69)	(87.31, 84.69, 80.93)	(59.32, 61.27, 60.28)	(47.68, 46.93, 48.66)
3	(110.91, 114.36, 113.24)	(86.91, 84.09, 90.67)	(64.91, 61.97, 68.35)	(46.28, 45.37, 49.67)
4	(99.32, 114.59, 117.23)	(96.32, 84.30, 81.68)	(64.07, 62.39, 65.62)	(50.62, 48.32, 43.28)
5	(115.36, 110.67, 117.36)	(87.96, 92.42, 91.07)	(62.71, 63.44, 60.21)	(47.23, 46.35, 47.94)
6	(112.09, 117.89, 110.39)	(84.36, 87.43, 89.61)	(57.33, 64.35, 61.26)	(50.36, 50.20, 48.69)
7	(106.23, 109.65, 109.84)	(85.31, 84.36, 83.49)	(60.16, 65.28, 61.41)	(46.93, 44.37, 46.31)
8	(105.36, 109.64, 108.94)	(91.25, 86.30, 82.42)	(63.68, 62.97, 60.58)	(45.36, 50.19, 47.71)
(**b**)				
**Number of Experiments**	**Fundamental Cross-Correlation Error** **(*x*, *y*, *z*) mm**	**Quadratic Correlation Error** **(*x*, *y*, *z*) mm**	**Cubic Correlation Error** **(*x*, *y*, *z*) mm**	**Generalized Cubic Correlation Error** **(*x*, *y*, *z*) mm**
1	(107.94, 112.38, 111.72)	(93.21, 95.68, 82.03)	(61.24, 69.93, 64.07)	(43.48, 47.66, 42.88)
2	(110.86, 116.29, 101.32)	(81.21, 83.19, 81.09)	(58.83, 60.99, 62.37)	(45.39, 45.71, 47.57)
3	(115.08, 116.54, 111.34)	(82.33, 82.91, 94.87)	(58.36, 66.32, 66.38)	(44.95, 47.34, 48.06)
4	(100.93, 111.04, 115.37)	(90.24, 87.37, 89.22)	(69.21, 65.44, 61.37)	(50.82, 44.99, 47.33)
5	(117.96, 114.05, 112.11)	(92.38, 87.63, 93.57)	(59.17, 64.43, 58.17)	(43.81, 48.66, 42.94)
6	(103.52, 100.77, 114.58)	(82.64, 92.31, 82.68)	(59.62, 62.85, 69.46)	(48.76, 48.73, 48.55)
7	(101.76, 113.36, 115.61)	(81.98, 89.04, 86.69)	(58.47, 67.32, 68.93)	(42.36, 48.77, 44.14)
8	(103.24, 112.42, 104.74)	(88.74, 82.36, 86.72)	(65.37, 67.48, 67.74)	(42.38, 49.43, 45.02)
(**c**)				
**Number of Experiments**	**Fundamental Cross-Correlation Error** **(*x*, *y*, *z*) mm**	**Quadratic Correlation Error** **(*x*, *y*, *z*) mm**	**Cubic Correlation Error** **(*x*, *y*, *z*) mm**	**Generalized cubic correlation error** **(*x*, *y*, *z*) mm**
1	(107.34, 113.37, 104.11)	(96.04, 86.26, 87.58)	(59.49, 66.58, 59.98)	(43.32, 47.29, 48.75)
2	(104.92, 109.88, 113.07)	(86.76, 89.24, 92.23)	(58.01, 65.14, 63.52)	(45.16, 49.39, 44.31)
3	(114.52, 111.22, 108.04)	(81.13, 86.33, 95.35)	(60.12, 66.82, 59.75)	(40.78, 43.92, 43.26)
4	(100.99, 102.37, 109.95)	(96.09, 89.50, 86.03)	(60.15, 59.63, 68.35)	(50.05, 45.77, 46.23)
5	(114.66, 102.61, 110.96)	(82.24, 96.29, 90.67)	(64.81, 60.71, 62.83)	(45.03, 40.22, 44.29)
6	(116.47, 106.93, 100.79)	(81.32, 90.06, 87.11)	(58.70, 64.79, 68.15)	(50.36, 50.27, 49.19)
7	(111.65, 103.89, 111.30)	(86.99, 89.12, 88.09)	(65.36, 66.08, 59.91)	(43.33, 48.87, 45.17)
8	(107.66, 108.54, 110.32)	(92.65, 88.06, 89.11)	(59.96, 64.43, 69.03)	(40.16, 43.29, 44.32)
(**d**)				
**Number of Experiments**	**Fundamental Cross-Correlation Error** **(*x*, *y*, *z*) mm**	**Quadratic Correlation Error** **(*x*, *y*, *z*) mm**	**Cubic correlation Error** **(*x*, *y*, *z*) mm**	**Generalized Cubic Correlation Error** **(*x*, *y*, *z*) mm**
1	(111.86, 107.53, 109.03)	(90.28, 85.29, 89.77)	(69.03, 66.98, 64.81)	(43.61, 47.27, 44.52)
2	(115.71, 108.24, 106.73)	(84.23, 86.04, 91.51)	(58.71, 66.47, 63.21)	(41.34, 47.06, 42.43)
3	(101.38, 104.76, 109.94)	(82.95, 87.62, 91.57)	(59.19, 63.83, 69.26)	(42.97, 46.59, 49.06)
4	(111.32, 114.09, 117.88)	(91.77, 89.63, 85.25)	(63.67, 62.49, 63.27)	(44.06, 46.83, 42.72)
5	(116.57, 110.29, 115.24)	(85.82, 92.55, 95.37)	(59.36, 64.18, 69.15)	(43.36, 42.01, 40.97)
6	(100.69, 107.51, 111.14)	(89.27, 93.57, 96.45)	(58.67, 60.76, 65.35)	(48.36, 43.25, 46.88)
7	(102.36, 108.71, 109.61)	(84.81, 88.31, 93.08)	(61.11, 65.37, 69.77)	(43.28, 41.65, 40.91)
8	(109.42, 117.54, 118.01)	(81.28, 85.31, 89.72)	(66.66, 65.50, 60.72)	(43.83, 49.14, 50.29)

**Table 4:** (**a**) Location results of Partial Discharge Source 1 (Corona Discharge type). (**b**) Location results of Partial Discharge Source 2 (Surface Discharge type).

(a)			
Experiment Number	Position of Partial Discharge Source 1 (*x*, *y*, *z*) mm	Actual Value (*x*, *y*, *z*) mm	Error (*x*, *y*, *z*) mm
1	(300, 330, 160)	(255.68, 289.42, 118.69)	(44.32, 40.58, 41.31)
2	(200, 370, 220)	(246.66, 328.32, 269.88)	(46.66, 41.68, 49.88)
3	(260, 410, 170)	(307.55, 368.46, 226.53)	(47.55, 41.54, 56.53)
4	(355, 260, 260)	(305.32, 216.64, 308.72)	(49.68, 43.36, 48.72)
5	(410, 175, 220)	(456.23, 130.69, 269.54)	(46.23, 44.31, 49.54)
6	(455, 290, 360)	(404.82, 340.27, 315.48)	(50.18, 50.27, 44.52)
7	(520, 340, 160)	(463.32, 295.63, 226.91)	(56.68, 44.37, 46.91)
8	(600, 295, 390)	(558.64, 345.19, 346.39)	(41.36, 50.19, 43.61)
(**b**)			
**Experiment Number**	**Position of Partial Discharge** **Source 2 (*x*, *y*, *z*) mm**	**Actual Value (*x*, *y*, *z*) mm**	**Error (*x*, *y*, *z*) mm**
1	(195, 420, 170)	(152.69, 375.64, 228.93)	(42.31, 44.36, 58.93)
2	(320, 290, 300)	(368.46, 336.55, 256.97)	(48.46, 46.55, 43.03)
3	(400, 450, 130)	(356.28, 404.62, 176.06)	(43.72, 45.38, 40.06)
4	(230, 380, 400)	(279.16, 334.68, 354.92)	(49.16, 45.32, 45.08)
5	(290, 300, 410)	(246.37, 346.26, 368.61)	(43.63, 46.26, 41.39)
6	(340, 460, 330)	(294.79, 412.66, 378.04)	(45.21, 47.34, 48.04)
7	(420, 480, 290)	(468.31, 432.94, 346.38)	(48.31, 47.06, 56.38)
8	(720, 400, 190)	(674.95, 352.62, 234.57)	(45.05, 47.38, 44.57)

## Data Availability

The data will be provided upon request.

## Appendix A

To maximize the accuracy of time delay estimation in low signal-to-noise ratio environments, it is necessary to identify an optimal weighting function that maximizes the signal-to-noise ratio (Deflection Coefficient) at the peak of the generalized triple cross-correlation function.

According to Equation (3), the frequency-domain expression of the generalized triple cross-correlation can be expressed as the sum of the principal signal component, S(ω), and the noise component, N(ω):

(A1) $$W_{RRR}(j\omega )=S(\omega )\cdot e^{-j\omega \tau }+N(\omega )$$

Here, the principal signal component is defined as $S(\omega )=G_{G_{ss}^{*3}}(j\omega )$, while the noise component N(ω) comprises the remaining terms in Equation (3) (i.e., $\sum G_{G_{u_{i}u_{i}}^{*3}}$ and $G_{G_{VV}^{*3}}$, etc.).

Following the application of the weighting filter H(jω), the time-domain output SNR, ${\left(SNR\right)}_{out}$, is defined as the ratio of the square of the output peak to the output noise variance:

(A2) $${\left(SNR\right)}_{out}=\frac{|E[R_{out}(\tau )]{|}^{2}}{Var[R_{out}(\tau )]}$$

According to Parseval’s theorem, the output peak signal power can be expressed as:

(A3) $$|E[R_{out}(\tau )]{|}^{2}={\left|\frac{1}{2\pi }\int_{-\infty }^{\infty }H(j\omega )S(\omega )d\omega \right|}^{2}$$

Under the assumption of low SNR, the variance of the output noise is primarily dominated by the noise power spectrum. Assuming that the frequency components are uncorrelated, the variance can be approximated as:

(A4) $$Var[R_{out}(\tau )]\approx \frac{1}{2\pi }\int_{-\infty }^{\infty }|H(j\omega ){|}^{2}|N(\omega ){|}^{2}d\omega$$

Consequently, the expression for the SNR becomes:

(A5) $${\left(SNR\right)}_{out}=\frac{1}{2\pi }\frac{{\left|\int_{-\infty }^{\infty }H(j\omega )S(\omega )d\omega \right|}^{2}}{\int_{-\infty }^{\infty }|H(j\omega ){|}^{2}|N(\omega ){|}^{2}d\omega }$$

To determine the H(jω) that maximizes the above expression, we apply the Schwarz Inequality:

(A6) $${\left|\int A(\omega )B(\omega )d\omega \right|}^{2}\leq \int |A(\omega ){|}^{2}d\omega \cdot \int |B(\omega ){|}^{2}d\omega$$

Let A(ω)=H(jω)N(ω), and let $B(\omega )=\frac{S(\omega )}{N(\omega )}$. Substituting them into the inequality yields:

(A7) $${\left|\int H(j\omega )S(\omega )d\omega \right|}^{2}\leq \int |H(j\omega )N(\omega ){|}^{2}d\omega \cdot \int {\left|\frac{S(\omega )}{N(\omega )}\right|}^{2}d\omega$$

The equality holds if and only if $A(\omega )=k\cdot B^{*}(\omega )$ (where k is a constant), at which point the SNR is maximized. This implies:

(A8) $$H(j\omega )N(\omega )=k\cdot \frac{S^{*}(\omega )}{N^{*}(\omega )}\Rightarrow H(j\omega )=k\frac{S^{*}(\omega )}{|N(\omega ){|}^{2}}$$

Mapping this to the specific variables defined in this paper, the signal term corresponds to $S(\omega )=G_{G_{ss}^{*3}}$, and the noise term corresponds to $|N(\omega )|\approx \sum G_{G_{u_{i}u_{i}}^{*3}}\cdot G_{G_{VV}^{*3}}$ (aligning with the form of the denominator in Equation (4)). By neglecting the constant k and adopting a as a normalization coefficient, we obtain the optimal weighting function for maximizing SNR:

(A9) $$H(j\omega )=\frac{a\cdot G_{G_{ss}^{*3}}}{{\left(\sum_{i=1}^{N}G_{G_{u_{i}u_{i}}^{*3}}\cdot G_{G_{VV}^{*3}}\right)}^{2}}$$

This corresponds exactly to the form presented in Equation (4). The derivation demonstrates that the squared term in the denominator arises from the minimization of noise variance, while the numerator stems from the matching of signal energy, thereby theoretically ensuring the algorithm’s optimality under low SNR conditions.
